# The Effects of Irisin on Nω-Nitro-L-arginine Methyl Ester Hydrochloride-Induced Hypertension in Rats

**DOI:** 10.4274/balkanmedj.galenos.2019.2019.5.113

**Published:** 2019-11-05

**Authors:** Nurettin Aydoğdu, Özlem Yalçınkaya Yavuz, Ebru Taştekin, Pınar Tayfur, Oktay Kaya, Nihayet Kandemir

**Affiliations:** 1 Department of Physiology, Trakya University School of Medicine, Edirne, Turkey; 2 Department of Pathology, Trakya University School of Medicine, Edirne, Turkey

**Keywords:** Hypertension, irisin, kidney, nitric oxide, oxidative stress, rats

## Abstract

**Background:**

The cause of about 95% of hypertension, an important public health problem, is unknown. Intensive studies are underway to understand the physiopathology of hypertension. Irisin, a newly discovered hormone, has been reported to dilate vascular smooth muscle and lower blood pressure acutely.

**Aims:**

To investigate the effects of chronic irisin treatment on blood pressure and renal functions in a hypertension model established by nitric oxide synthase inhibition by treatment with Nω-nitro-L-arginine methyl ester hydrochloride.

**Study Design:**

Animal experimentation.

**Methods:**

Male Sprague−Dawley rats were divided into four groups (n=8). Control and irisin groups received an intravenous saline injection, hypertension and hypertension + irisin (hypertension + irisin) groups received 1.5 mg/100 g Nω-nitro-L-arginine methyl ester hydrochloride. Nω-nitro-L-arginine methyl ester hydrochloride (150 mg/L) was added to the drinking water of rats in groups hypertension and hypertension + irisin for three weeks. In the second week of the experiment, irisin (50 nmol/day) was given to rats in groups irisin and hypertension + irisin, and saline was administered to rats in groups control and hypertension for two weeks through subcutaneously placed osmotic minipumps. Blood pressure was measured by the tail-cuff plethysmography method. On the twenty-first day of the experiment, 24-hour urine, blood, and both kidneys of the rats were collected.

**Results:**

The hypertension group had elevated systolic, diastolic, and mean arterial blood pressure values compared with the control group, with decreased glutathione levels in tissue and serum, but an increase in serum oxidized glutathione level (p<0.05). Histopathologically, increased tubular injury, cast formation, glomerular sclerosis, and peritubular fibrosis levels were observed (p<0.05). Irisin treatment did not cause any significant change in blood pressure, renal functions, and injury scores. However, renal nitric oxide levels significantly increased, and endothelial nitric oxide synthase immunoreactivity was determined to be reduced (p<0.05).

**Conclusion:**

Treatment with chronic irisin at a physiological dose does not reduce blood pressure in an experimental model of hypertension. In different models of experimental hypertension, the effects of irisin administration at different doses and at different periods should be thoroughly investigated.

Hypertension (HT) is a health condition that is common in society and causes severe mortality and morbidity because of its effects on “so-called” target organs, including the kidneys. HT is one of the major risk factors for the development of cardiovascular disease, with nearly 95% of cases being essential HT. Epidemiologic studies in developed and developing countries in recent years showed that the prevalence of essential HT is on an increasing trend in most countries. In many industrialized countries, the prevalence is reported to vary from 25% to 55% (1). Further, in parallel with its increasing prevalence, the repayment costs for medications used to treat target organ injury and comorbid diseases has increased organizations’ expenditures for this disease. The prevention of HT will reduce morbidity, mortality, and expenditures for diagnostic and treatment procedures, and associated target organ injury and complications. Studies to determine the mediators that play roles in the pathophysiology of HT and research into their treatment efficacy continue to increase (2).

Nitric oxide synthase (NOS) enzymes are commonly found in the body and have a variety of functions. NOS enzymes exist as three different isoforms. These are neuronal NOS, inducible NOS, and endothelial NOS. The nitric oxide (NO) required for physiologic functions is synthesized by endothelial NOS and neuronal NOS. inducible NOS causes longer-term effects and higher amounts of NO synthesis compared with those of other isoforms, and this increases the severity of the physiologic effects of NO. In the inflammatory process, nearly all cells produce high levels of NO mediated by inducible NOS. The excess NO transforms into the stable end products of this radical of nitrite and nitrate or competes with superoxide dismutase enzymes to create peroxynitrite because of the interaction with superoxide radicals (3,4).

The new hormone of irisin, so-called myokine, was discovered by Boström et al. (5) in 2012. It has a molecular weight of 12,587 kDa and is a polypeptide consisting of 112 amino acids. Irisin is released after exercise by skeletal muscles and is reported to transform white fat cells into brown fat cells (6). Currently, irisin has been shown to be expressed at various levels in skeletal muscles, heart muscle, fat tissue, the liver, the brain, bone, the pancreas, the ovaries, and the kidneys (7). In addition, irisin plays a protective role against endothelial injury and is reported to reduce blood pressure (8). It has been shown that acute intravenous injection of irisin reduces blood pressure in a dose-dependent manner (0.1, 1, and 10 µg/kg). In this study, it was reported that physiological irisin concentrations (48 and 240 nmol/L) did not decrease blood pressure (9). In contrast, the pharmacological concentrations of irisin in the in vitro study were reported to be 50 to 100 nmol/L (10). However, the possible effect of irisin, especially long-term irisin treatment, on blood pressure and HT, remains unclear.

In this study, we hypothesized that long-term administration of low-dose irisin given at physiological concentrations would reduce blood pressure. We aimed to investigate irisin levels and the effects chronic irisin therapy administered at physiological doses on blood pressure, oxidative stress, the vascular NOS mechanism, and renal function in an experimental HT model induced by Nω-nitro-L-arginine methyl ester hydrochloride (L-NAME).

## MATERIALS AND METHODS

### Animal ethics

A total of 32 adult male (330-390 g) Sprague−Dawley rats were randomly divided into four equal groups. Animals were obtained from Trakya University Faculty of Medicine Experimental Animals Unit. All animals were housed in an environment with 12 hour light-12 hour dark cycles, 55% humidity, and 21±2 °C. They were fed with standard feed. Ethical approval was obtained by Trakya University Animal Experiments Local Ethics Committee (TUHADYEK-2015/34).

### Experimental design

**Control (C) group:** Intravenous physiologic serum (1 mL/kg) was administered via the tail vein, and then rats in this group were given tap water ad libitum for the duration of the experiment. After one week, minipumps were replaced with ALZET 2ML2 osmotic minipumps containing saline for two weeks.

**Irisin (I) group:** Intravenous physiologic serum (1 mL/kg) was administered via the tail vein, and then rats in this group were given tap water ad libitum for the duration of the experiment. After one week, minipumps were replaced with ALZET 2ML2 osmotic minipumps containing irisin (Phoenix Pharmaceuticals, USA; 50 nmol/day) for two weeks. The selected dose of irisin was based on the previous study (9,10).

**HT group:** For the HT group, a bolus dose was given IV (1.5 mg/100 g of body weight via the tail vein) followed by L-NAME ad libitum in the drinking water (150 mg/L) for the duration of the experiment. After one week, osmotic minipumps were replaced with ALZET 2ML2 minipumps containing saline for two weeks (11).

**HT + I group:** In this group, a bolus dose was given IV (1.5 mg/100 g of body weight via the tail vein) followed by L-NAME ad libitum in the drinking water (150 mg/L) for the duration of the experiment. After one week, minipumps were replaced with ALZET 2ML2 osmotic minipumps containing irisin (50 nmol/day) for two weeks.

### Preparation and implantation of osmotic minipumps

The osmotic minipumps (ALZET 2ML2, Cupertino, CA, USA) were set to 5 µL/hr infusion rate for 14 days. Rats were anesthetized with intramuscular 10 mg/kg xylazine and 50 mg/kg ketamine. Osmotic pumps were surgically implanted in rats under the skin, at the back of the neck, and between the two scapulae.

### Blood pressure measurements

Blood pressure measurements of rats in all groups were taken with indirect tail-cuff plethysmography (MAY NIBP250, Ankara, Turkey). Measurements were taken on the first, seventh, fourteenth, and twenty-first days. For each animal, a total of five measurements were made at 1 minute intervals. The highest and lowest measurements were ignored, and the mean was calculated from the three remaining measurements. The mean blood pressure was calculated with the formula;

Mean blood pressure= diastolic blood pressure + (systolic blood pressure–diastolic blood pressure)/3

### Termination of experiment and collection of tissue, serum, and urine samples

Rats in all groups were held in metabolic cages for the final 24 hours of the experiment and end blood pressure measurements were taken after collecting urine samples. Rats were euthanized by exsanguination after taking blood and both kidneys under 10 mg/kg xylazine and 50 mg/kg ketamine anesthesia. Half of the right kidney was placed in 10% formalin solution for histopathologic examination, while the other portions were placed in liquid nitrogen for analysis and stored at -80 °C. Blood and urine samples were centrifuged at +4 °C at 3000 rpm for 10 minutes in a cooled centrifuge and stored at -80 °C.

### Biochemical analysis

Serum urea, creatinine, sodium, potassium levels and alanine aminotransferase, aspartate aminotransferase and creatine kinase activities, and urine creatinine and sodium measurements were performed with an autoanalyzer (Abbott Architect c16000, USA) in Trakya University Health Research and Application Center Laboratory. Creatinine clearance was calculated using the standard clearance formula. The fractional excretion of sodium was calculated as a percentage.

Irisin levels in serum and urine were determined with the ELISA method (Elabscience, Cat. no: E-EL-R1104). Ox-glutathione (GSH) levels in renal tissue were measured according to procedures specified by the kit (Mybiosource, Cat no: MBS752665), while Red-GSH levels were also measured with a kit (Mybiosource, Cat no: MBS724319).

The end product of lipid peroxidation of malondialdehyde was measured spectrophotometrically with the color change (pink) due to the interaction with thiobarbituric acid in a hot and acidic medium (12,13). Results are expressed as nmol/g. Nitrate and nitrite were measured according to the method described by Cortas and Wakid (14). Estimation of protein amounts was performed according to the Lowry method (15).

### Histological analysis

For the light microscopic investigation, kidneys were cut in the sagittal plane and were fixed in 10% formalin, submerged in paraffin blocks and cut into 5-micron thick sections. Sections were stained with the hematoxylin-eosin method for light microscopy. Preparations were semiquantitatively assessed for glomerular necrosis, glomerular basal membrane thickening, mesangial matrix widening, tubular hydropic degeneration, and tubular dilatation. Accordingly, pathological changes are scored as 0 no pathology, +1 focal, +2 moderate focal, +3 multifocal, and +4 diffuse (16).

### Immunohistochemical analysis

For immunohistochemical analysis, formalin-fixed and, in sections prepared from paraffin-embedded tissue, 4 mm thick sections were used. Tissue sections were taken to electrostatically charged slides and dried at 70 °C for at least one hour. The entire immunohistochemical staining process, including deparaffinization and antigen expression, was performed in a fully automated immunohistochemistry staining device (Ventana BenchMark Ultra, Ventana Medical System, Tucson, AZ). A biotinylated, HRP multimer-based, hydrogen peroxide substrate and a ready-to-use kit containing the 3, 3-diaminobenzidine tetrahydrochloride chromogen were used for the process. The immunohistochemical antibody [irisin (Bioss FNDC5 BS-4886-R), inducible NOS (Spring REF E3744), and endothelial NOS (Neomarkers RB-9279-P)] panel used for the diagnosis and differential diagnosis varies according to the cases and cytokeratin. In the opposite dye staining apparatus, the process was terminated by dehydration of the sections, defined by hematoxylin and bluing solution, clarifying with xylene, and closing with a coverslip. All immunopositive cells were evaluated in a random portion of 10 large growth areas (x200). Cells stained for each case were scored and given in percent. Staining prevalence was graded as 0 (0%-5%), 1 (6%-24%), 2 (25%-49%), 3 (50%-74%), and 4 (75%). The staining intensity was graded as 0 (negative), 1 (mild), 2 (moderate), and 3 (strong). Two values were multiplied to obtain an immunoreactivity score of 0-300, as described in our previous study (17).

### Statistical analysis

Results are shown as mean ± standard deviation or median (interquartile range). Statistical analysis was performed using SPSS 18.0 software for Windows. The normal distribution of the quantitative data was assessed by the Shapiro Wilk test. Although there were four groups in the study, no comparison was made between the two groups. A Student *t*-test was used instead of ANOVA designs to compare the variables showing normal distribution between C-I, C-HT, and HT-HT + I groups. A Mann-Whitney U test was used to compare the variables not showing a normal distribution. P<0.05 was accepted as the limit of statistical significance.

## RESULTS

### Measurement of oxidative stress markers in renal tissue

Oxidative stress was assessed using oxidative stress markers, including malondialdehyde and GSH in renal tissue. Reduced GSH in serum (p<0.05) was significantly lower in the HT group compared with the C group and oxidized GSH (p<0.01) was significantly higher in the HT group compared with the C group. Reduced GSH (p<0.05) and oxidized GSH (p=0.05) in tissue were significantly lower in the HT group compared with the C group. The mean and standard deviation data for variables in all groups are shown in Table 1.

### Measurement of nitric oxide concentration in serum, renal tissue, and urine

NO in renal tissue (p<0.05) was significantly higher in the H + I group compared with the HT group. The mean and standard deviation data for variables in all groups are shown in Table 2.

### Measurement of irisin levels in serum and urine

There was no significant difference in irisin levels in serum between the groups. Urine irisin (p<0.05) levels were significantly higher in the HT group compared with those in the C group. The mean and standard deviation data for variables in all groups are shown in Table 3.

### Biochemical results

Serum sodium levels were significantly lower in the HT group compared with those in the C group and were significantly increased in the HT + I group compared with those in the HT group. Serum alanine aminotransferase levels were significantly higher in the HT + I group compared with those in the HT group. The mean and standard deviation data for biochemical variables in all groups are shown in Table 4.

### Results of blood pressure measurements

**Figure 1:**
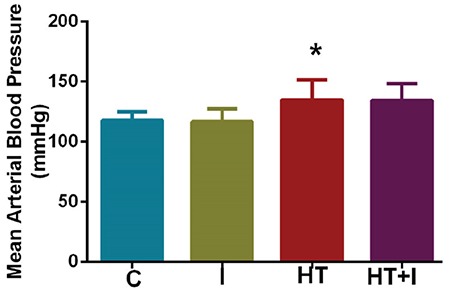
Mean arterial blood pressure values for all groups. *: p<0.05 C: control; I: irisin; HT: hypertension; HT + I: hypertension + irisin group

In the HT group, systolic, diastolic, and mean arterial pressure values were observed to increase significantly with L-NAME administration, whereas heart rate decreased significantly (p<0.05). The blood pressure values for all groups are shown in Figure 1.

### Histopathologic and immunoreactivity results

**Figure 2:**
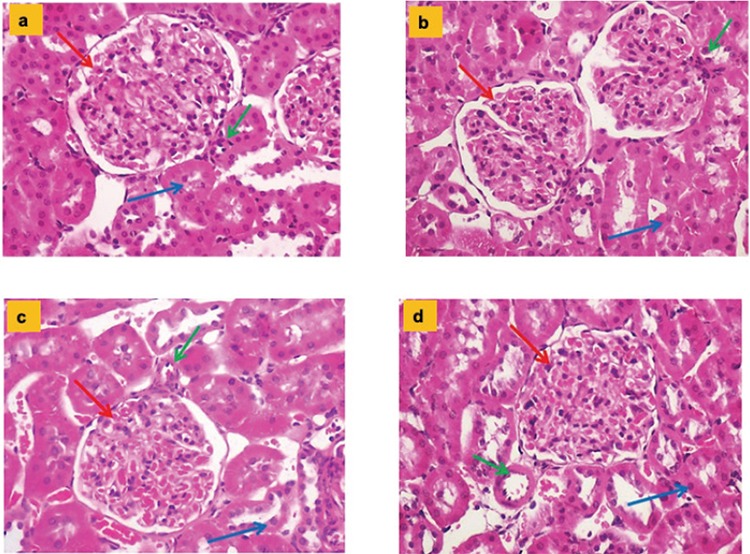
**a-d.** Hematoxylin and eosin-stained sections of rat kidneys (Hematoxylin and eosin x200). Glomeruli (red arrows) and tubules (blue arrows) with regular structure were observed. In the Hypertension and Hypertension + irisin groups, mild levels of peritubular fibrosis and sclerosis findings in glomeruli were observed. Control group (a); Irisin group (b); Hypertension group (c); Hypertension + irisin group (d).

Glomeruli and tubules with regular structure were observed. Pathologic findings were not observed in the vein at the vascular pole (common results for C, I, HT, and HT + I groups are shown with the green arrow). In the HT and HT + I groups, mild levels of peritubular fibrosis and sclerosis findings in the glomeruli were observed; however, apparent histopathologic changes secondary to HT were observed (p<0.01). The histopathologic findings for all the groups are shown in Table 5 and Figure 2.

**Figure 3:**
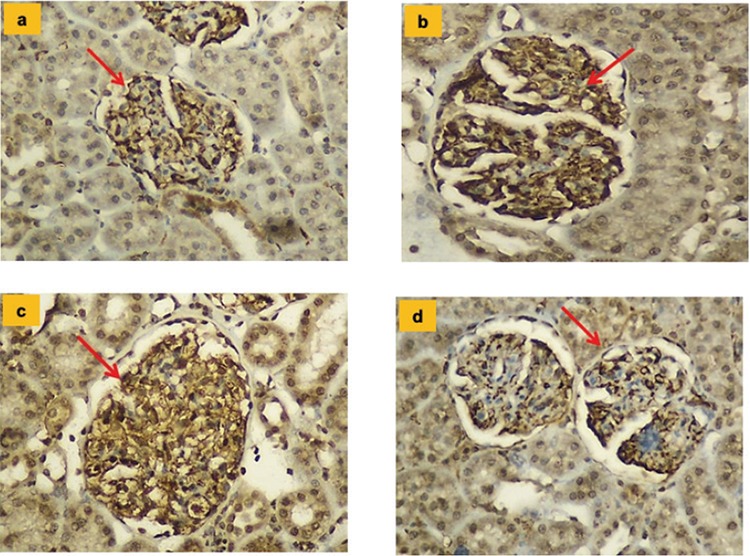
**a-d.** Glomerular immunostaining for endothelial nitric oxide synthase in different groups (x200). In all groups, widespread strong staining of glomerular capillaries (red arrow) and weak staining of proximal tubules were observed. When the Hypertension and Hypertension + irisin groups are compared, the Hypertension + irisin group was observed to have a significantly reduced level of staining (p<0.01). Control group (a); Irisin group (b); Hypertension group (c); Hypertension + irisin group (d).

**Figure 4:**
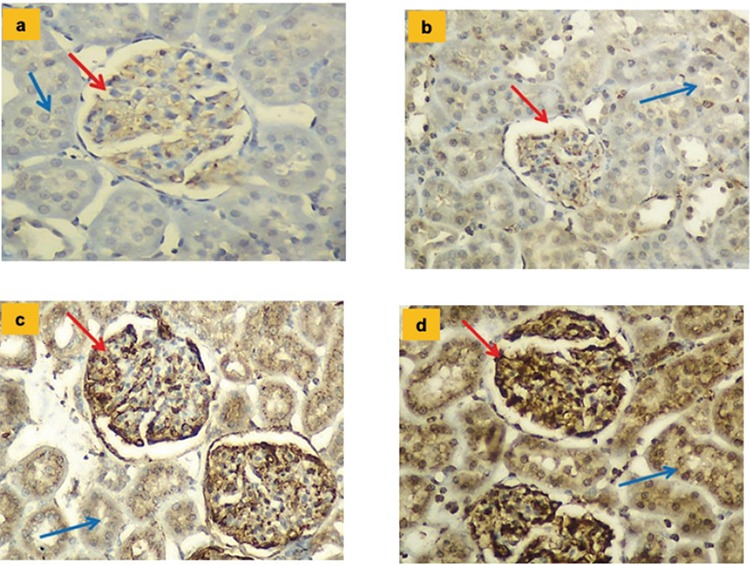
**a-d.** Glomerular immunostaining for inducible nitric oxide synthase in different groups (x200). While the control and B groups were observed to have focal and mild staining [glomeruli (red arrow), tubules (blue arrow)], there was widespread and strong staining in the Hypertension and Hypertension + irisin groups (p<0.01). Control group (a); Irisin group (b); Hypertension group (c); Hypertension + irisin group (d).

**Figure 5:**
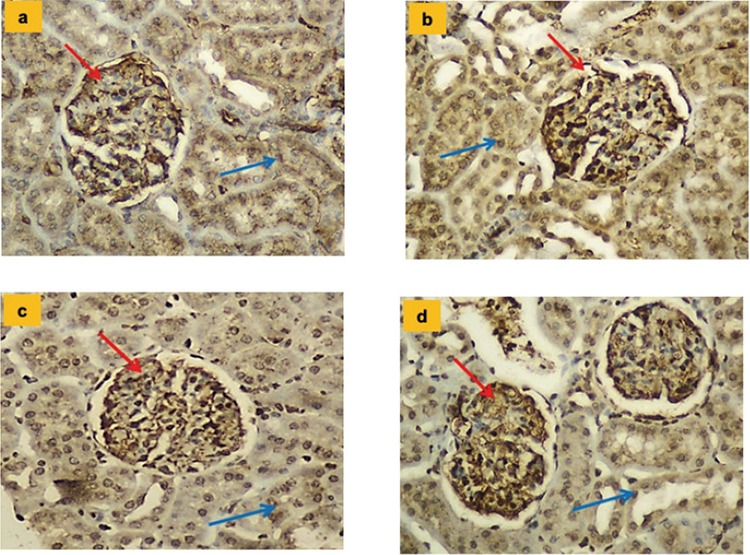
**a-d.** Glomerular immunostaining for neuronal nitric oxide synthase in different groups (x200). All groups viewed had similar widespread staining of glomerular capillaries (red arrow) and proximal tubules (blue arrow). Control group (a); Irisin group (b); Hypertension group (c); Hypertension + irisin group (d).

**Figure 6:**
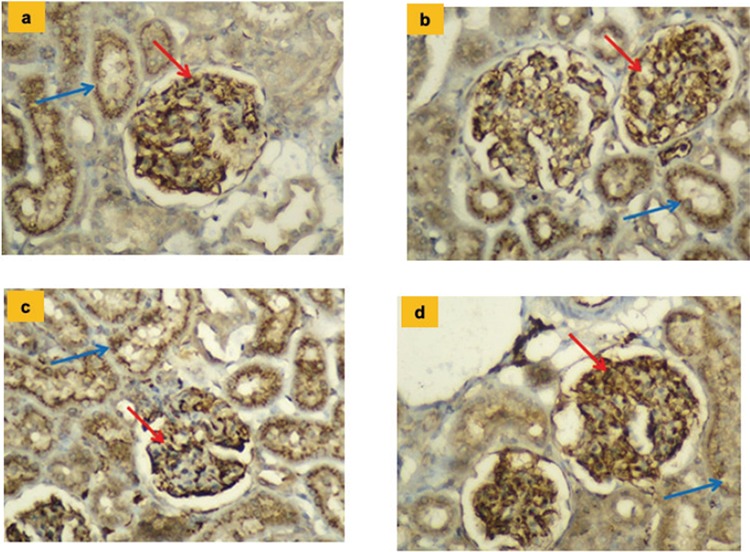
**a-d.** Glomerular immunostaining for neuronal irisin in different groups (x200). All groups had staining with similar features in the glomerular capillaries (red arrow) and the distal tubules (blue arrow). Control group (a); Irisin group (b); Hypertension group (c); Hypertension + irisin group (d).

The histopathologic results and mean values for endothelial NOS, inducible NOS, and neuronal NOS values belonging to the groups and tissue irisin immunoreactivity results are shown in Table 6 and Figure 3, 4, 5, 6.

## DISCUSSION

In this study, by using an L-NAME-induced experimental HT model, we obtained the following results in the HT group:

I) There was no significant difference in serum irisin levels and irisin immunoreactivity of renal tissue. The increase in the urine irisin levels was not significant.

II) Histopathological examination of the kidney showed significantly higher tubular damage, glomerular sclerosis, peritubular fibrosis, and cast formation.

III) No significant changes in renal function markers were observed. However, serum Na+ levels decreased significantly.

IV) Serum and renal glutathione levels were significantly reduced. A significant increase in serum oxidized glutathione levels was observed.

V) Although there was a significant increase in renal endothelial NOS and inducible NOS immunoreactivity, there was no significant difference in neuronal NOS immunoreactivity.

Treatment with irisin did not cause any significant change in the high mean arterial blood pressure. Serum Na+ levels decreased significantly in the HT + I group. In addition, irisin treatment caused a significant increase in renal NO levels.

HT presents a significant risk factor for subclinical renal injury in humans and animals. In rats, HT development with the NOS inhibitor L-NAME is reported to cause glomerular injury and injury to the interstitial area in the kidneys due to vasoconstriction. Administration of L-NAME at different doses and durations to experimental animals induces HT. This model is commonly used because of observations of renal complications linked to HT, similar to those in humans (18,19).

Histopathological examination of the kidney showed a significant increase in tubular damage, peritubular fibrosis, glomerular sclerosis, and caste levels in the HT group. The effect of increased blood pressure on the occurrence of renal damage in different types of HT and the relationship between elevated blood pressure and renal dysfunction is different. It is reported that detailed studies are needed to define the relationship between elevated blood pressure, renal damage, and renal dysfunction. Although renal injury was significantly increased in our study, no statistically significant difference was observed in renal dysfunction. These findings are consistent with the results of previous studies (19,20). An increase in systemic blood pressure produces a natriuretic and diuretic effect. As tubular reabsorption is impaired in HT, increased Na+ excretion has been reported (20). The significant decrease in serum Na levels in the HT group of our study may be the result of tubular damage. Our findings in this regard are consistent with previous study results (20). In our study, no significant difference was observed in glomerular functions; L-NAME may be the result of insufficient dosing and treatment duration.

Oxidative stress plays an important role in the pathophysiology of HT. Endogenous and exogenous antioxidants show an antihypertensive effect. Free radicals, such as superoxide, reduce the bioavailability of nitric oxide. The main function of endothelial NOS is the production of NO that regulates vasodilation. However, the lack of oxidation of L-arginine and tetrahydrobiopterin, separation of the L-arginine-NO pathway reduces NO formation and causes endothelial NOS-mediated superoxide production. Superoxide combines with NO synthesized by endothelial NOS to form peroxynitrite, which promotes endothelial NOS uncoupling and ROS production (21,22). HT is also known to be associated with disruption of glutathione metabolism. Reduced glutathione levels have an important function as they protect proteins and membrane lipids from oxidation. Our protocol aimed at investigating whether oxidative stress parameters in the HT group were significantly decreased and reduced GSH levels in serum and renal tissue, whereas serum oxide GSH levels were determined to be increased. This data is in accordance with the results of studies inducing HT by administering L-NAME to rats at different doses and duration (22). In our study, the increase in renal tissue malondialdehyde levels, an end product of lipid peroxidation used as a marker of oxidative stress, was not significant. This result does not agree with the results of some studies in the literature (22). We have no plausible explanation for this except that the L-NAME dose and administration duration were different.

When NO levels of the control and the HT groups were compared, there was no significant difference. However, the immunohistochemistry results for endothelial NOS and inducible NOS activity showed a significant increase in the HT group compared with the control group. Administration of L-NAME is thought to block NO synthesis, which we assume is because of higher NOS activity in certain tissues *ex vivo* and higher levels of NOS gene expression. Paradoxically, the effect of L-NAME on NOS expression *in vivo* appears to vary with treatment duration and the tissue being examined (23). Administration of L-NAME at 40 mg/kg/day for four weeks was reported to cause an increase in endothelial NOS and inducible NOS expression in cavernous tissue in Sprague–Dawley rats and increased endothelial NOS in the heart and the kidneys of Wistar rats. However, it did not affect the expression of endothelial NOS in brain tissue (24). In addition, the same dose of L-NAME did not affect endothelial NOS or inducible NOS expression in the aorta of Wistar rats after five weeks of treatment, whereas seven weeks treatment increased e the expression of endothelial NOS in the heart but reduced its expression in the brain (25). In our study, increased expression of NOS may be the result of endothelial NOS uncoupling. In addition, the blood pressure-lowering effect in the peripheral irisin is reported to be very brief.

Recently, it has been reported that irisin may play an essential role in the pathophysiology of cardiovascular diseases, including HT (9,26,27). In these studies, Zhang et al. (26) administered irisin centrally to the third ventricle at doses of 0.625-2.5 μg/rat and recorded increased blood pressure and cardiac contractility. In contrast, injection of peripheral intravenous high-dose irisin (2-8 µg/rat) has been reported to reduce blood pressure in both control and spontaneously hypertensive rats. It has been shown to cause dilatation of mouse mesenteric artery, *in vitro*. In addition, it is reported that the blood pressure-lowering effect of peripheral irisin is very short-lived. This study is important since it demonstrates that different routes of irisin administration may have different effects (26). In another study, bolus injections of irisin (two minutes) were shown to reduce blood pressure in spontaneously hypertensive rats in a dose-dependent (0.1, 1, and 10 µg/kg) manner. In this study, it was reported that low doses or physiological irisin concentrations (48 and 240 nmol/L) did not decrease blood pressure and did not dilate the mesenteric artery of Wistar Kyoto rats.

The authors indicated that there was no direct vasodilator effect, although irisin reduces blood pressure after high-dose intravenous administration (9). When the doses used in animal studies are converted to those prescribed to humans, these doses can be very high. Therefore, we wanted to investigate the long-term effects of a physiological dose of irisin (50 nmol/day). In the current study, it was determined that irisin treatment at a physiology dose did not cause any significant change in blood pressure. The reason for this may be that an insufficient physiological dose of irisin was administered. Therefore, we believe that further studies should be performed using pharmacological doses of irisin in different experimental models of HT. Chen et al. (27) showed that circulating irisin levels increased in patients with HT compared with the control group. They proposed that increased circulating levels of irisin might be associated with HT and stroke due to HT. In our study, we observed a significant increase in renal tissue NO levels in the HT+I group. According to the study by Zhu et al. (28), irisin administration to diabetic rats causes NO to increase and improves endothelial function. The results of this study are similar to our findings.

There are some limitations to our study. First, the physiological dose of long-term irisin was used. Therefore, the effects of the pharmacological dose could not be determined. Second, the dose and duration of L-NAME that were administered to induce HT did not impair renal function. Third, the effects of irisin were only investigated in L-NAME-induced experimental HT models.

In conclusion, we found that long-term administration of physiological concentrations of irisin does not ameliorate blood pressure in an L-NAME-induced HT model. In contrast, irisin treatment increased serum sodium levels. In addition, renal NO levels and endothelial NOS immunoreactivity increased. According to the results of the current study, irisin may have advantages and disadvantages regarding blood pressure. Further studies are needed to determine whether there are different effects at different doses of irisin on the NO-mediated mechanism and whether irisin has therapeutic potential in different models of HT.

## Figures and Tables

**Table 1:**
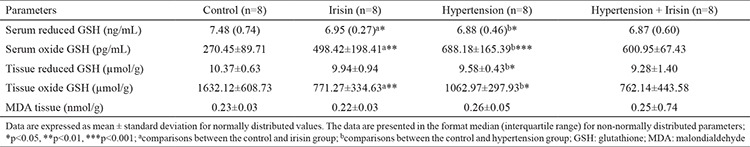
Oxidative stress levels in renal tissue of all groups

**Table 2:**
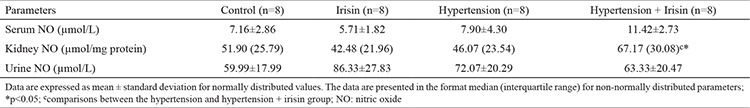
Serum, renal tissue, and urine nitric oxide concentrations of all groups

**Table 3:**
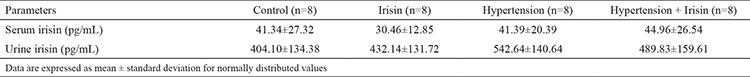
Serum and urine irisin levels of all groups

**Table 4:**
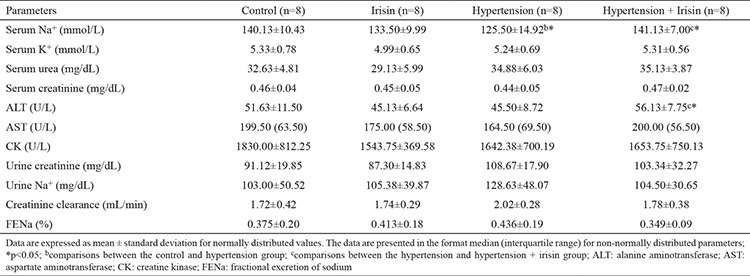
Biochemical parameters of all groups

**Table 5:**
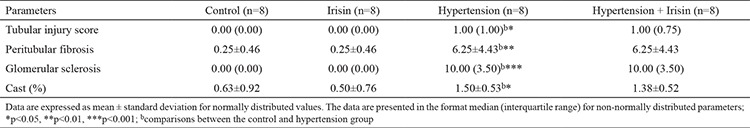
Histopathologic values of all groups

**Table 6:**
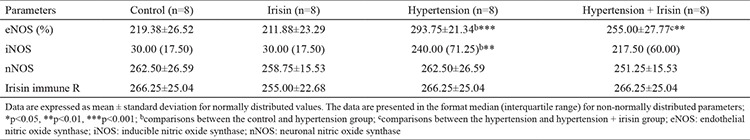
Immunohistochemical levels of all groups
